# Risk factors for wet macular degeneration: a systematic review, with novel insights from the Scottish Heart Health Extended Cohort

**DOI:** 10.1186/s12886-025-03868-5

**Published:** 2025-02-10

**Authors:** Catherine A. Fitton, Madeleine M. R. Quigley, Jill J. F. Belch

**Affiliations:** Division of Molecular & Clinical Medicine, Mailbox 1, University of Dundee, Ninewells Hospital, Dundee, DD1 9SY UK

**Keywords:** Macular degeneration, Risk factors, Cigarette smoking

## Abstract

**Background:**

Age-related macular degeneration (AMD) is a major cause of vision loss worldwide. This study aimed to assess risk factors for wet AMD by two methods: assessing risk factors measured in the Scottish Heart Health Extended Cohort (SHHEC), and to systematically review the literature.

**Methods:**

Eighteen thousand one hundred seven volunteers were recruited to SHHEC between 1984–1995, with risk factor data collected on recruitment. Hospital records were linked to study data up to 2017 and survival analysis was used to analyse risk factors and wet AMD. Literature published between 2000–2023 was searched for studies assessing risk factors for wet AMD, resulting in 5,503 papers. Following review, 7 studies were included in the systematic review.

**Results:**

Within the SHHEC data, 231 cases of wet AMD were reported. Increasing age (Hazard Ratio (HR) 10.51; 99% Confidence Interval (CI) 4.78–23.11) and smoking (HR 1.67; 99% CI 1.17–2.38) were significantly associated with an increased risk of wet AMD. Increased dietary intake of vitamin K (HR 0.56; 99% CI 0.34–0.94) was associated with a decreased risk of wet AMD.

According to a systematic review, smoking, high Body mass index, heavy alcohol intake, increased systolic blood pressure, increased pulse pressure, and high levels of C-reactive protein and serum triglycerides in the blood may be associated with an increased risk of wet AMD. However, the studies provide mixed evidence and no conclusive results.

**Conclusion:**

We have demonstrated that increasing age and smoking are high-risk factors for the development of wet AMD, while vitamin K is associated with a reduced risk of wet AMD.

**Supplementary Information:**

The online version contains supplementary material available at 10.1186/s12886-025-03868-5.

## Introduction

Age-related macular degeneration (AMD) is a disease producing progressive visual impairment caused by late-onset neurodegeneration of the photoreceptor–retinal pigment epithelial complex [1]. The pathology of AMD is a result of the interaction between metabolic, functional, genetic, and environmental factors, such as oxidative damage from light exposure and oxidative stress [1]. AMD is a major source of vision loss world-wide, accounting for vision loss in 10% of people older than 65 years and 25% of people older than 75 years [2]. In the UK alone, AMD cases were estimated to be 608,213 in 2010 and were expected to rise to 755,867 in 2020 [3]. There are two main types of AMD: geographic atrophy or dry AMD; and neovascularisation or wet AMD. Dry AMD is a slow process of vision loss and is not currently treatable, whereas wet AMD is a rapid deterioration of vision, partially treatable by injections of anti-vascular endothelial growth factor (anti-VEGF) into the affected eye [3].

Several risk factors contributing to AMD have been documented and can be categorised into nonmodifiable and modifiable risk factors. Nonmodifiable risk factors include increasing age [4], sun exposure [5, 6], race, family history of AMD and eye colour [7]. While modifiable lifestyle factors include smoking [8–10], heavy alcohol intake [11] and body weight [12]. Smoking is a well explored risk factor and has long been agreed to be a preventable cause of AMD, however the evidence for other modifiable risk factors is not fully robust [13, 14].

We aimed to assess wet AMD incidence in relation to risk factors measured in the Scottish Heart Health Extended Cohort (SHHEC), and to systematically review the literature for evidence for the importance or otherwise of these risk factors in AMD.

## Methods

### Ethics and permissions

This study was approved by the National Health Service Scotland Public Benefit and Privacy Panel for Health and Social Care. Reference 1516–0578/2223–0068.

For the original clinical study ethical approval was received in 1984 from the Tayside Ethics Committee, approval no 39/84.

### SHHEC participants and inclusion/exclusion

This study utilizes the data from the Scottish Heart Health Extended Cohort (SHHEC), which has been described in detail previously [15]. It combined the Scottish Heart Health Study (SHHS) [16, 17] and the Scottish MONICA study [18], where volunteers were randomly recruited across 23 districts of Scotland between 1984–1987, and from Edinburgh and North Glasgow over 1986–1995 respectively. Both men and women, aged 40–59 were recruited into SHHS, and aged 25–75 were recruited for MONICA, totalling 18,107 for SHHEC. Demographic details and blood samples were taken at recruitment [15]. Participants had their body weight, height, waist circumference and blood pressure measured at recruitment by a member of the recruitment team. Smoking habits, and eating habits were recorded via a self-administered questionnaire. A food frequency questionnaire (FFQ) was used to determine the eating habits of participants. In the FFQ, 99 items were assessed, with one frequency category (once a week). Permission was given for long term follow up via record linkage.

### AMD data

Participants in SHHEC had their Community Health Index (CHI) numbers linked with the Scottish Morbidity Record 01 (SMR01), which registered acute hospital admissions, and National Records of Scotland (NRS), which recorded deaths. We identified relevant wet AMD cases by using the following International Classification of Diseases 10th edition (ICD10) code: H35.3 Degeneration of macular and posterior pole. In the UK, wet AMD is treated with anti-VEGF injections as an acute hospital visit. Other types of AMD are not seen in hospital and are dealt with in secondary care (General Practice surgeries and opticians), so only wet AMD cases are recorded in the SMR01 data. As there have been several versions of ICD codes used over the years, eDRIS converted all past ICD codes to the most recent version when they provided the data to researchers, meaning only one version of ICD was required (ICD10 2016 version).

### Statistics

Cox proportional hazard models were used to estimate hazard ratios with 99% confidence intervals (HR 99% CI), which allowed for different enrolment time of participants. Survival was counted to the first qualifying event (first anti-VEGF injection for wet AMD). Loss to follow up occurred through death. Data were adjusted for age, sex, smoking status, alcohol intake and body weight.

## Methods for literature review

### Systematic review data sources and study selection

A systematic review protocol was prepared as per standard Preferred Reporting Items for Systematic Reviews and Meta-Analyses (PRISMA) guidelines [19] and registered with the International Prospective Register of Systematic Reviews (PROSPERO) 02/07/2020 [20]. The databases Web of Science, the Cochrane Database of Systematic Review (CDSR) library, and Scopus (Elsevier) were searched from January 2000 to July 2023 inclusive (last search conducted on 4th July 2023), using the search terms (contained in keywords, title or abstract): risk and macular degeneration. As only human studies were considered, exclusion terms were tailored towards removing animal studies, using the following search terms (contained in keywords, title or abstract): animal, rat, mice and mouse. Wildcard symbols, truncation, combinations of search terms using Boolean operators, and alternative spellings were used.

Prospective cohort studies of any size, which reported adult subjects of any ethnicity and age, from any country, with an outcome of age-related macular degeneration were included. We included studies that reported any potential risk factors which were also measured in the SHHEC cohort (supplementary Table 1).

The reference lists from identified papers were scanned for other relevant studies. Narrative and systematic reviews, meta-analyses, retrospective or cross-sectional studies, studies published only as abstracts, letters with no data, or conference proceedings, animal studies and editorials were excluded. Initial screening of titles was carried out to identify potentially relevant studies, followed by screening of abstracts and then by full paper review. All titles and abstracts were independently evaluated by two reviewers (CAF, MMRQ) and any discrepancies were discussed and agreed. When discrepancies arose, a third reviewer was consulted (JJFB).

### Quality and bias assessment

Quality and bias assessments were conducted by the two independent reviewers (CAF, MMRQ) utilising the Newcastle Ottawa Scale for cohort studies [21] to rate the studies as poor, good or excellent. Tables detailing these are included as supplements (supplemental file 2).

### Study review and data extraction

**Fig. 1:**
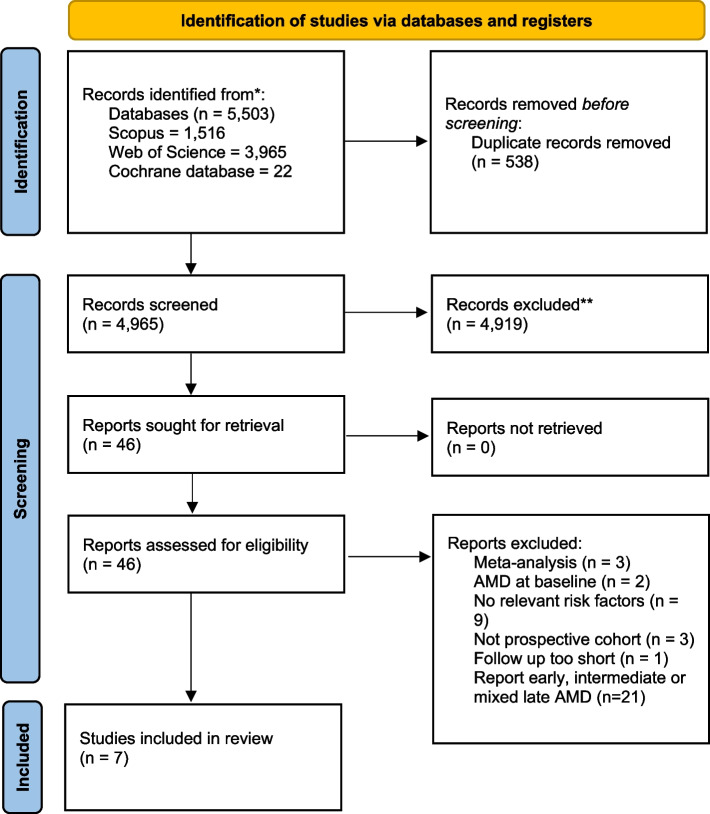
PRISMA flow chart for systematic review

The information extracted from studies was as follows: appropriateness of case and control recruitment, inclusion and exclusion criteria clearly stated, appropriate validation of cases, appropriate analysis, and sufficient follow up information. Initially a meta-analysis was planned, however, as the qualifying studies were few (*n* = 7), and due to lack of study homogeneity between study predictors and outcomes, a narrative synthesis was conducted instead Fig. 1.

## Results

### SHHEC data

Of the 18,107 subjects enrolled in SHHEC, 231 were treated for wet AMD in hospital over the follow up period. Participants were followed for an average of 30.3 years (range of 9–34 years). Supplemental Table 1 reports the variables measured in the SHHEC cohort on recruitment with the mean, median and missing values. Due to the number of variables reported in this study, the results have been split into five categories, detailed below.

#### Personal factors

Older age (aHR 10.51; 99% CI 4.78–23.11) was associated with a significantly increased risk in wet AMD (Table 1). Height, body weight and other personal risk factors were not significantly associated with either an increased or decreased risk of wet AMD (Table 1).

**Table 1:** Analyses of personal risk factors as predictors of macular degeneration within SHHEC. Results are presented as Hazard Ratios (HR) with 99% Confidence Intervals (CI), and P values. Two models presented: no adjustment; age, sex, smoking, BMI and alcohol adjusted. Continuous data grouped as quartiles, or as specifically defined groups (i.e. education etc.)

Variable	Grouping	Macular degeneration		Adjusted for + age + sex + smoking + BMI + alcohol	
		HR (99% CI)	*P* value	HR (99% CI)	*P* value
Age (years)	Q1 ≤ 43.0	Reference		Reference	
	Q2 = 43.1–49.7	4.20 (1.91–9.25)	< 0.001	4.04 (1.83–8.92)	< 0.001
	Q3 = 49.8–55.8	8.01 (3.73–17.20)	< 0.001	8.01 (3.71–17.28)	< 0.001
	Q4 ≥ 55.9	10.65 (4.89–23.22)	< 0.001	10.51 (4.78–23.11)	< 0.001
Sex	M = 8,862	Reference		Reference	
	F = 9,245	1.39 (0.98–1.97)	0.015	1.37 (0.93–2.02)	0.036
Education (years)	Group 1 < 10	Reference		Reference	
	Group 2 = 10	0.51 (0.33–0.80)	< 0.001	1.27 (0.77–2.10)	0.218
	Group 3 11–12	0.62 (0.35–1.10)	0.032	1.72 (0.94–3.15)	0.020
	Group ≥ 13	0.48 (0.29–0.79)	< 0.001	1.29 (0.75–2.21)	0.224
Family Cardiovascular disease history	N = 12,717	Reference		Reference	
	Y = 5,390	1.01 (0.69–1.47)	0.951	0.96 (0.66–1.41)	0.793
Scottish Index of Multiple Deprivation (SIMD)	Q1 ≤ 10.9	Reference		Reference	
	Q2 = 11.0–22.1	1.66 (1.05–2.62)	0.005	1.47 (0.92–2.34)	0.033
	Q3 = 22.2–40.2	1.46 (0.90–2.38)	0.046	1.20 (0.73–1.97)	0.344
	Q4 ≥ 40.3	1.34 (0.79–2.29)	0.155	1.11 (0.64–1.94)	0.631
Height (m)	Q1 ≤ 1.58	Reference		Reference	
	Q2 = 1.59–1.65	0.83 (0.53–1.29)	0.272	1.02 (0.65–1.62)	0.898
	Q3 = 1.66–1.72	0.56 (0.34–0.94)	0.004	0.88 (0.48–1.62)	0.598
	Q4 ≥ 1.73	0.71 (0.44–1.13)	0.054	1.33 (0.66–2.69)	0.292
Weight (kg)	Q1 ≤ 61	Reference		Reference	
	Q2 = 62–69	0.91 (0.56–1.48)	0.629	0.89 (0.52–1.53)	0.576
	Q3 = 70–79	0.92 (0.58–1.48)	0.661	0.90 (0.47–1.71)	0.668
	Q4 ≥ 80	0.92 (0.57–1.48)	0.662	0.88 (0.36–2.11)	0.697
BMI (kg/m^2^)	Q1 ≤ 22.9	Reference		Reference	
	Q2 = 23.0–25.3	1.16 (0.71–1.89)	0.384	1.06 (0.65–1.73)	0.703
	Q3 = 25.4–27.9	1.17 (0.71–1.91)	0.486	0.97 (0.58–1.61)	0.793
	Q4 ≥ 28.0	1.32 (0.81–2.16)	0.139	1.12 (0.68–1.84)	0.554
BMI (kg/m^2^)	1kg/m2 increase	1.03 (0.99–1.07)	0.053	1.02 (0.98–1.06)	0.223
Waist circumference (cm)	Q1 ≤ 75.9	Reference		Reference	
	Q2 = 76.0–84.9	0.91 (0.26–3.20)	0.853	0.57 (0.12–2.80)	0.433
	Q3 = 85.0–93.9	0.38 (0.07–2.14)	0.151	0.31 (0.03–2.85)	0.177
	Q4 ≥ 94.0	0.67 (0.16–2.84)	0.478	0.39 (0.02–6.70)	0.427

#### Smoking factors

Smoking at time of recruitment (aHR 1.67; 99% CI 1.17–2.38), smoking 11–20 cigarettes per day (aHR 1.66; 99% CI 1.01–2.71) and smoking more than 20 cigarettes per day (aHR 1.86; 99% CI 1.19–2.91) were associated with an increased risk of wet AMD (Table 2). Markers for immediate past smoking such as levels of cotinine, thiocyanate and expired carbon monoxide in the blood were not significantly associated with an increased risk of AMD.

**Table 2:** Analyses of smoking risk factors as potential predictors of macular degeneration within SHHEC. Results are presented as Hazard Ratios (HR) with 99% Confidence Intervals (CI), and P values. Two models presented: no adjustment; age, sex, smoking, BMI and alcohol adjusted. Continuous data grouped as quartiles, or as specifically defined groups (i.e. education etc.)

Variable	Grouping	Macular degeneration		Adjusted for + age + sex + smoking + BMI + alcohol	
		HR (99% CI)	*P* value	HR (99% CI)	*P* value
Current smoker	*N* = 9,759	Reference		Reference	
	Y = 8,348	1.30 (0.93–1.83)	0.047	1.67 (1.17–2.38)	< 0.001
Cigarettes/day	Group 1 = 0	Reference		Reference	
	Group 2 = 1–5	0.84 (0.36–1.96)	0.602	0.93 (0.36–2.41)	0.855
	Group 3 = 6–10	1.63 (0.73–3.67)	0.119	1.91 (0.85–4.30)	0.041
	Group 4 = 11–20	1.43 (0.88–2.34)	0.057	1.66 (1.01–2.71)	0.008
	Group 5 > 20	1.30 (0.84–2.00)	0.120	1.86 (1.19–2.91)	< 0.001
Expired Carbon Monoxide (ppm)	Q1 ≤ 1.9	Reference		Reference	
	Q2 = 2.0–2.9	1.11 (0.63–1.97)	0.639	1.11 (0.62–1.99)	0.654
	Q3 = 3.0–14.9	1.15 (0.67–1.96)	0.509	1.16 (0.65–2.06)	0.510
	Q4 ≥ 15.0	1.51 (0.87–2.62)	0.055	1.69 (0.81–3.51)	0.065
Cotinine (ng/ml)	Q1 ≤ 0.49	Reference		Reference	
	Q2 = 0.5–4.2	0.99 (0.56–1.75)	0.951	1.02 (0.56–1.86)	0.927
	Q3 = 4.3–233.0	0.92 (0.51–1.64)	0.695	1.07 (0.48–2.38)	0.826
	Q4 ≥ 233.1	1.60 (0.94–2.74)	0.024	2.01 (0.78–5.14)	0.056
Thiocyanate (µlmol/L)	Q1 ≤ 32.9	Reference		Reference	
	Q2 = 33.0–50.6	1.30 (0.75–2.26)	0.216	1.34 (0.76–2.36)	0.189
	Q3 = 50.7–110.0	1.10 (0.61–1.98)	0.686	1.07 (0.54–2.12)	0.805
	Q4 ≥ 110.1	2.01 (1.17–3.43)	< 0.001	2.14 (0.95–4.81)	0.015

#### Cardiovascular factors

A systolic blood pressure of > 144mmHg (HR 1.63; 99% CI 1.01–2.62), a pulse pressure of above 59mmHg (HR 1.67; 99% CO 1.01–2.75), LDL > 3.2mmol/L (HR 2.16; 99% CI 1.21–3.87) and an NT-pro BNP of above 24.2pg/ml (HR 2.01; 99% CI 1.03–3.93) were associated with an increased risk of wet AMD before adjustment for known risk factors. Following adjustment for age, sex, body weight, smoking and alcohol intake, these associations disappeared (Table 3). No other blood pressure or lipid parameters measured showed any significant association.

**Table 3:** Analyses of cardiovascular risk factors as potential predictors of macular degeneration within SHECC. Results are presented as Hazard Ratios (HR) with 99% Confidence Intervals (CI), and P values. Two models presented: no adjustment; age, sex, smoking, BMI and alcohol adjusted. Continuous data grouped as quartiles, or as specifically defined groups (i.e. education etc.)

Variable	Grouping	Macular degeneration		Adjusted for + age + sex + smoking + BMI + alcohol	
		HR (99% CI)	*P* value	HR (99% CI)	*P* value
Systolic Blood Pressure (mmHg)	Q1 ≤ 117	Reference		Reference	
	Q2 = 118–128	1.19 (0.73–1.93)	0.354	0.99 (0.61–1.63)	0.979
	Q3 = 129–143	1.02 (0.62–1.68)	0.910	0.76 (0.46–1.27)	0.175
	Q4 ≥ 144	1.63 (1.01–2.62)	0.009	0.90 (0.54–1.50)	0.581
Systolic BP	10mmHg increase	1.07 (0.98–1.16)	0.061	0.95 (0.86–1.04)	0.131
Diastolic Blood Pressure (mmHg)	Q1 ≤ 72	Reference		Reference	
	Q2 = 73–80	1.13 (0.71–1.82)	0.493	0.96 (0.59–1.55)	0.828
	Q3 = 81–88	1.13 (0.70–1.83)	0.503	0.91 (0.55–1.49)	0.615
	Q4 ≥ 89	0.89 (0.53–1.50)	0.568	0.66 (0.39–1.14)	0.052
Diastolic BP	10mmHg increase	0.99 (0.86–1.14)	0.865	0.91 (0.78–1.06)	0.110
Pulse pressure (mmHg)	Q1 ≤ 40	Reference		Reference	
	Q2 = 41–48	1.40 (0.86–2.26)	0.075	1.26 (0.77–2.06)	0.224
	Q3 = 49–58	1.28 (0.78–2.11)	0.199	0.98 (0.59–1.63)	0.911
	Q4 ≥ 59	1.67 (1.01–2.75)	0.009	0.95 (0.56–1.61)	0.799
Pulse rate (beats/min)	Q1 ≤ 67	Reference		Reference	
	Q2 = 68–75	1.39 (0.81–2.38)	0.118	1.41 (0.81–2.46)	0.108
	Q3 = 76–83	1.25 (0.72–2.18)	0.305	1.27 (0.72–2.26)	0.283
	Q4 ≥ 84	1.29 (0.76–2.19)	0.210	1.33 (0.77–2.29)	0.176
Total Cholesterol (mmol/L)	Q1 ≤ 5.3	Reference		Reference	
	Q2 = 5.4–6.1	1.13 (0.66–1.91)	0.559	0.90 (0.52–1.53)	0.597
	Q3 = 6.2–7.0	1.11 (0.65–1.90)	0.612	0.71 (0.41–1.25)	0.119
	Q4 ≥ 7.1	1.49 (0.90–2.47)	0.042	0.77 (0.45–1.31)	0.203
HDL (mmol/L)	Q1 ≤ 1.15	Reference		Reference	
	Q2 = 1.16–1.41	0.87 (0.49–1.54)	0.547	0.83 (0.46–1.48)	0.411
	Q3 = 1.42–1.71	1.03 (0.60–1.77)	0.846	0.87 (0.49–1.56)	0.587
	Q4 ≥ 1.72	1.07 (0.63–1.82)	0.719	0.87 (0.48–1.60)	0.591
Non-HDL (mmol/L)	Q1 ≤ 3.8	Reference		Reference	
	Q2 = 3.9–4.6	1.17 (0.70–1.97)	0.426	0.87 (0.51–1.47)	0.494
	Q3 = 4.7–5.5	1.04 (0.60–1.78)	0.869	0.66 (0.38–1.15)	0.055
	Q4 ≥ 5.6	1.27 (0.76–2.14)	0.230	0.67 (0.39–1.16)	0.063
LDL (mmol/L)	Q1 ≤ 1.8	Reference		Reference	
	Q2 = 1.9–2.4	1.26 (0.66–2.42)	0.356	1.04 (0.54–2.00)	0.870
	Q3 = 2.5–3.1	1.24 (0.64–2.40)	0.392	0.88 (0.45–1.72)	0.632
	Q4 ≥ 3.2	2.16 (1.21–3.87)	< 0.001	1.38 (0.76–2.49)	0.168
Triglycerides (mmol/L)	Q1 ≤ 1.0	Reference		Reference	
	Q2 = 1.1–1.5	1.17 (0.70–1.94)	0.432	0.95 (0.56–1.60)	0.788
	Q3 = 1.6–2.3	1.33 (0.80–2.19)	0.145	0.96 (0.56–1.65)	0.859
	Q4 ≥ 2.4	1.31 (0.78–2.19)	0.180	1.02 (0.58–1.81)	0.915
Apolipoprotein-A (g/L)	Q1 ≤ 1.2	Reference		Reference	
	Q2 = 1.3–1.4	1.64 (0.92–2.90)	0.026	1.54 (0.87–2.74)	0.054
	Q3 = 1.5–1.6	1.59 (0.90–2.81)	0.038	1.37 (0.76–2.47)	0.172
	Q4 ≥ 1.7	1.34 (0.75–2.40)	0.199	1.04 (0.55–1.94)	0.887
Apolipoprotein-B (g/L)	Q1 ≤ 0.8	Reference		Reference	
	Q2 = 0.9–1.0	0.90 (0.53–1.52)	0.598	0.67 (0.39–1.15)	0.058
	Q3 = 1.1–1.2	1.14 (0.68–1.90)	0.524	0.74 (0.44–1.26)	0.147
	Q4 ≥ 1.3	1.14 (0.68–1.93)	0.513	0.65 (0.38–1.13)	0.046
Lipoprotein a (mg/dL)	Q1 ≤ 5.1	Reference		Reference	
	Q2 = 5.2–11.2	1.03 (0.60–1.78)	0.872	0.97 (0.56–1.67)	0.873
	Q3 = 11.3–28.5	0.98 (0.56–1.72)	0.941	0.86 (0.50–1.51)	0.502
	Q4 ≥ 28.6	1.32 (0.79–2.21)	0.166	1.22 (0.73–2.05)	0.323
Homocysteine (µmol/L)	Q1 ≤ 11.3	Reference		Reference	
	Q2 = 11.4–13.5	1.16 (0.68–1.97)	0.486	1.00 (0.58–1.72)	0.999
	Q3 = 13.6–16.5	1.27 (0.75–2.16)	0.249	0.97 (0.56–1.68)	0.889
	Q4 ≥ 16.6	1.51 (0.89–2.57)	0.047	1.16 (0.67–2.01)	0.494
HsTroponin I (pg/mL)	Q1 ≤ 1.8	Reference		Reference	
	Q2 = 1.9–3.9	1.22 (0.66–2.26)	0.399	0.92 (0.49–1.72)	0.733
	Q3 = 4.0–6.0	1.40 (0.78–2.51)	0.144	1.04 (0.57–1.88)	0.880
	Q4 ≥ 6.1	1.75 (0.98–2.15)	0.014	1.16 (0.63–2.13)	0.536
NT-pro BNP (pg/mL)	Q1 ≤ 24.2	Reference		Reference	
	Q2 = 24.3–49.0	2.01 (1.03–3.93)	0.007	1.65 (0.84–3.25)	0.058
	Q3 = 49.1–93.5	2.22 (1.15–4.31)	0.002	1.58 (0.79–3.13)	0.089
	Q4 ≥ 93.6	2.68 (1.37–5.23)	< 0.001	1.57 (0.77–3.20)	0.103

#### Diabetes mellitus

No diabetes mellitus factors measured were significantly associated with an increased or decreased risk of developing wet AMD such as glucose, insulin and C-Peptide (Table 4).

**Table 4:** Analyses of diabetes risk factors as potential predictors of macular degeneration within SHHEC. Results are presented as Hazard Ratios (HR) with 99% Confidence Intervals (CI), and P values. Two models presented: no adjustment; age, sex, smoking, BMI and alcohol adjusted. Continuous data grouped as quartiles, or as specifically defined groups (i.e. education etc.)

Variable	Grouping	Macular degeneration		Adjusted for + age + sex + smoking + BMI + alcohol	
		HR (99% CI)	*P* value	HR (99% CI)	*P* value
Diabetes	*N* = 17,777	Reference		Reference	
	Y = 330	2.36 (0.74–7.57)	0.058	2.09 (0.65–6.74)	0.104
Glucose (mmol/L)	Q1 ≤ 4.31	Reference		Reference	
	Q2 = 4.32–4.75	1.23 (0.72–2.10)	0.325	1.22 (0.71–2.10)	0.336
	Q3 = 4.76–5.27	0.89 (0.50–1.59)	0.608	0.88 (0.49–1.57)	0.558
	Q4 ≥ 5.28	1.77 (1.06–2.95)	0.004	1.62 (0.96–2.74)	0.017
Insulin (µU/mL)	Q1 ≤ 3.8	Reference		Reference	
	Q2 = 3.9–6.4	0.87 (0.52–1.46)	0.481	0.92 (0.54–1.55)	0.674
	Q3 = 6.5–11.7	0.72 (0.42–1.26)	0.131	0.80 (0.45–1.42)	0.318
	Q4 ≥ 11.8	1.00 (0.60–1.67)	0.999	1.12 (0.65–1.94)	0.585
c-peptide (mg/mL)	Q1 ≤ 1.5	Reference		Reference	
	Q2 = 1.6–2.2	1.05 (0.60–1.85)	0.815	0.97 (0.55–1.72)	0.889
	Q3 = 2.3–3.4	1.55 (0.92–2.63)	0.031	1.45 (0.85–2.48)	0.075
	Q4 ≥ 3.5	1.24 (0.71–2.16)	0.330	1.11 (0.62–1.99)	0.655

#### Inflammation and immunity

No inflammation or immunity factors were associated with a significant increased risk of wet AMD (Supplementary Table 2). This included standard markers for inflammation such as high sensitivity C-Reactive protein, fibrinogen and ferritin.

#### Liver and kidney

No liver and kidney risk factors measured were associated with a significant increase of wet AMD (Supplementary Table 3). This included gamma GGT, uric acid, creatinine, and cystatin C.

#### Coagulation

No coagulation factors measured, such as D-Dimer, were associated with a significant increase of wet AMD (Supplementary Table 4).

#### Nutrition factors

Dietary intake of vitamin K in the range of 43.9 – 60.7 mcg/day (HR 0.49; 99% CI 0.28–0.86) or ≥ 81.7 mcg/day (HR 0.56; 99% CI 0.34–0.94), and cereal fibre intake of 5.4–7.9g/day (HR 0.57 99% CI 0.34–0.97) was associated with a decreased risk of wet AMD (Table 5). Alcohol intake of 6.7 – 18.9g/day (HR 0.60 99% CI 0.38–0.95) was associated with a decreased risk of wet AMD before adjusting for known risk factors (Table 5). Following adjustment, the significance vanished. All other factors assessed, such as fat, protein, or carbohydrate intake, showed no association with the development of wet AMD (Table 5).

**Table 5:** Analyses of nutrients and wet AMD within SHHEC. Results are presented as Hazard Ratios (HR) with 99% Confidence Intervals (CI), and P values. Two models presented: no adjustment; age, sex, smoking, BMI and alcohol adjusted. Continuous data grouped as quartiles, or as specifically defined groups (i.e. education etc.)

Variable	Grouping	Macular degeneration		Adjusted for + age + sex + smoking + BMI + alcohol	
		HR (99% CI)	*P* value	HR (99% CI)	*P* value
Fat (g/day)	Q1 ≤ 63.7	Reference		Reference	
	Q2 = 63.8–79.9	0.96 (0.58–1.58)	0.944	1.00 (0.61–1.67)	0.761
	Q3 = 80.0–97.9	1.11 (0.69–1.81)	0.297	1.18 (0.72–1.94)	0.174
	Q4 ≥ 98.0	1.12 (0.68–1.83)	0.332	1.19 (0.72–1.99)	0.175
Saturated Fat (g/day)	Q1 ≤ 26.8	Reference		Reference	
	Q2 = 26.9–34.7	0.72 (0.42–1.23)	0.835	0.76 (0.44–1.32)	0.981
	Q3 = 34.8–43.9	1.17 (0.73–1.89)	0.566	1.23 (0.75–2.00)	0.379
	Q4 ≥ 44.0	1.12 (0.69–1.84)	0.568	1.15 (0.69–1.91)	0.374
Polyunsaturated fat (g/day)	Q1 ≤ 7.1	Reference		Reference	
	Q2 = 7.2–9.6	0.87 (0.54–1.42)	0.463	0.90 (0.55–1.48)	0.601
	Q3 = 9.7–13.1	0.89 (0.55–1.46)	0.555	1.00 (0.61–1.64)	0.996
	Q4 ≥ 13.2	0.94 (0.58–1.52)	0.744	1.11 (0.68–1.83)	0.581
Protein (g/day)	Q1 ≤ 67.4	Reference		Reference	
	Q2 = 67.5–79.5	0.90 (0.57–1.43)	0.570	0.94 (0.59–1.49)	0.729
	Q3 = 79.6–93.6	0.69 (0.42–1.13)	0.050	0.78 (0.47–1.29)	0.199
	Q4 ≥ 93.7	0.93 (0.58–1.48)	0.675	1.07 (0.65–1.75)	0.733
Carbohydrates (g/day)	Q1 ≤ 178.1	Reference		Reference	
	Q2 = 178.2–230.1	1.07 (0.68–1.67)	0.715	1.17 (0.74–1.84)	0.380
	Q3 = 230.2–293.1	0.86 (0.53–1.40)	0.425	0.99 (0.59–1.66)	0.976
	Q4 ≥ 293.2	0.88 (0.53–1.44)	0.494	1.17 (0.67–2.03)	0.472
Starch (g/day)	Q1 ≤ 105.9	Reference		Reference	
	Q2 = 105.9–140.0	1.09 (0.69–1.70)	0.638	1.16 (0.74–1.82)	0.405
	Q3 = 140.0–181.7	0.81 (0.50–1.32)	0.275	0.87 (0.52–1.46)	0.502
	Q4 ≥ 181.7	0.79 (0.48–1.30)	0.224	1.01 (0.58–1.76)	0.957
Sugar (g/day)	Q1 ≤ 61.7	Reference		Reference	
	Q2 = 61.8–84.7	0.91 (0.58–1.44)	0.598	0.99 (0.62–1.58)	0.962
	Q3 = 84.8–117.5	0.87 (0.54–1.41)	0.452	0.97 (0.59–1.60)	0.892
	Q4 ≥ 117.6	1.02 (0.63–1.65)	0.907	1.29 (0.77–2.16)	0.196
Alcohol (g/day)	Q1 = 0.0	Reference		Reference	
	Q2 = 0.1–6.6	0.69 (0.43–1.11)	0.044	0.74 (0.46–1.19)	0.101
	Q3 = 6.7–18.9	0.60 (0.38–0.95)	0.005	0.74 (0.46–1.19)	0.106
	Q4 ≥ 19.0	0.67 (0.42–1.07)	0.026	1.00 (0.59–1.69)	0.995
Cereal fibre (g/day)	Q1 ≤ 5.3	Reference		Reference	
	Q2 = 5.4–7.9	0.54 (0.32–0.91)	0.002	0.57 (0.34–0.97)	0.006
	Q3 = 8.0–11.4	0.82 (0.52–1.31)	0.281	0.85 (0.53–1.36)	0.364
	Q4 ≥ 11.5	0.85 (0.54–1.34)	0.362	0.87 (0.55–1.39)	0.446
Vegetable fibre (g/day)	Q1 ≤ 8.2	Reference		Reference	
	Q2 = 8.3–10.9	0.91 (0.57–1.46)	0.621	0.98 (0.61–1.58)	0.917
	Q3 = 11.0–13.8	0.85 (0.53–1.37)	0.383	0.97 (0.60–1.57)	0.865
	Q4 ≥ 13.9	0.77 (0.47–1.26)	0.167	0.85 (0.51–1.41)	0.405
Vitamin C (mg/d)	Q1 ≤ 36.3	Reference		Reference	
	Q2 = 36.4–51.0	0.76 (0.47–1.22)	0.129	0.80 (0.50–1.30)	0.241
	Q3 = 51.1–70.7	0.79 (0.49–1.27)	0.197	0.82 (0.51–1.32)	0.292
	Q4 ≥ 70.8	0.88 (0.55–1.41)	0.497	0.86 (0.53–1.39)	0.424
Total energy (kcal/d)	Q1 ≤ 1629.5	Reference		Reference	
	Q2 = 1629.6–1996.1	0.98 (0.62–1.56)	0.921	1.02 (0.64–1.63)	0.903
	Q3 = 1996.2–2443.3	0.98 (0.61–1.57)	0.909	1.12 (0.68–1.83)	0.569
	Q4 ≥ 2443.4	0.85 (0.51–1.41)	0.412	1.10 (0.62–1.94)	0.661
Dietary Cholesterol (mg/d)	Q1 ≤ 253.9	Reference		Reference	
	Q2 = 254.0–331.1	0.81 (0.49–1.35)	0.292	0.78 (0.46–1.30)	0.206
	Q3 = 331.2–425.0	1.15 (0.72–1.84)	0.448	1.10 (0.68–1.78)	0.596
	Q4 ≥ 425.1	1.03 (0.63–1.68)	0.884	1.05 (0.63–1.73)	0.816
Retinol (mcg/d)	Q1 ≤ 380.3	Reference		Reference	
	Q2 = 380.4–585.5	1.25 (0.76–2.05)	0.241	1.22 (0.74–2.00)	0.313
	Q3 = 585.6–880.7	0.88 (0.52–1.49)	0.531	0.84 (0.49–1.43)	0.393
	Q4 ≥ 880.8	1.26 (0.77–2.05)	0.232	1.13 (0.69–1.86)	0.529
Beta carotene (mg/d)	Q1 ≤ 1648.0	Reference		Reference	
	Q2 = 1648.1–3140.7	0.80 (0.49–1.32)	0.250	0.73 (0.45–1.21)	0.111
	Q3 = 3140.8–4573.4	0.82 (0.50–1.34)	0.305	0.75 (0.46–1.23)	0.136
	Q4 ≥ 4573.5	1.02 (0.64–1.63)	0.909	0.83 (0.52–1.34)	0.317
Alpha tocopherol (mg/d)	Q1 ≤ 4.0	Reference		Reference	
	Q2 = 4.1–5.4	0.83 (0.51–1.35)	0.329	0.86 (0.53–1.39)	0.409
	Q3 = 5.5–7.9	0.94 (0.58–1.52)	0.728	0.98 (0.60–1.61)	0.926
	Q4 ≥ 8.0	0.96 (0.59–1.55)	0.822	1.00 (0.61–1.63)	0.996
Linoleic acid (mg/d)	Q1 ≤ 5.2	Reference		Reference	
	Q2 = 5.3–7.3	0.76 (0.46–1.24)	0.144	0.81 (0.50–1.33)	0.273
	Q3 = 7.4–10.3	0.90 (0.56–1.47)	0.591	0.99 (0.60–1.62)	0.948
	Q4 ≥ 10.4	1.01 (0.63–1.61)	0.961	1.16 (0.72–1.88)	0.419
Iron (mg/d)	Q1 ≤ 9.3	Reference		Reference	
	Q2 = 9.4–11.5	0.99 (0.62–1.59)	0.976	1.02 (0.63–1.64)	0.918
	Q3 = 11.6–14.4	0.85 (0.52–1.39)	0.390	0.88 (0.54–1.46)	0.521
	Q4 ≥ 14.5	0.97 (0.60–1.56)	0.872	1.02 (0.63–1.67)	0.901
Vitamin K (mcg/d)	Q1 ≤ 43.8	Reference		Reference	
	Q2 = 43.9–60.7	0.46 (0.26–0.81)	< 0.001	0.49 (0.28–0.86)	0.001
	Q3 = 60.8–81.6	0.85 (0.53–1.35)	0.357	0.87 (0.55–1.39)	0.444
	Q4 ≥ 81.7	0.55 (0.33–0.92)	0.003	0.56 (0.34–0.94)	0.004
Trans fatty acids (g/d)	Q1 ≤ 4.6	Reference		Reference	
	Q2 = 4.7–6.1	0.97 (0.58–1.64)	0.89	0.97 (0.57–1.64)	0.877
	Q3 = 6.2–8.0	1.02 (0.61–1.70)	0.916	1.02 (0.61–1.72)	0.908
	Q4 ≥ 8.1	1.20 (0.73–1.97)	0.344	1.19 (0.72–1.97)	0.379
Vegetable trans Fatty Acids (g/d)	Q1 ≤ 1.7	Reference		Reference	
	Q2 = 1.8–2.4	1.10 (0.66–1.81)	0.638	1.18 (0.71–1.95)	0.404
	Q3 = 2.5–3.3	0.88 (0.51–1.50)	0.525	0.92 (0.54–1.58)	0.693
	Q4 ≥ 3.4	1.21 (0.73–2.00)	0.326	1.21 (0.72–2.02)	0.337
Animal trans Fatty Acids (g/d)	Q1 ≤ 1.9	Reference		Reference	
	Q2 = 2.0–3.4	1.08 (0.65–1.78)	0.705	1.03 (0.62–1.70)	0.897
	Q3 = 3.5–5.2	0.85 (0.50–1.45)	0.424	0.84 (0.49–1.45)	0.413
	Q4 ≥ 5.3	1.11 (0.67–1.85)	0.586	1.07 (0.64–1.80)	0.719

### Systematic review of risk factors

We reviewed literature published between the dates of January 2000-July 2023, for prospective studies, that assessed risk factors also assessed by the SHHEC study. The initial search resulted in 5,503 published articles, following duplication removal. Following title screening 578 articles remained, which was reduced further to 46 articles following abstract screening. Following full article screening, 7 articles were included in the final narrative review [14, 22–27]. Reasons for exclusion from the systematic review and meta-analysis included insufficient information reported, differences in risk factor measurement and differences in macular degeneration outcome. Corresponding authors of studies with insufficient data were contacted, with no responses providing the required data. We prospectively decided that three studies per risk factor (including SHHEC) minimum were required to assess data in a meta-analysis. The only risk factor with three studies, including SHHEC, was smoking. However, the two published studies did not include cohort numbers to allow meta-analysis. Figure 1 demonstrates the selection process. Supplementary Table 5 gives the study details for each paper. Risk factors reported were as follows: smoking, body weight, alcohol intake, diabetes mellitus status, C-reactive protein, systolic blood pressure, diastolic blood pressure, pulse pressure, serum cholesterol, serum HDL, LDL, triglycerides, beta-carotene, Vitamin B12.

#### Body weight

Two studies [24, 25] studied body weight as a potential risk factor for wet AMD, however both studies report different body weight measures. Jonasson et al. [24] reported body weight per kg/m2 increase and found no increased risk over the 5-year follow up period. Klein et al. [25] reported BMI, in categories of normal (22.0–29.9), low (< 22.0) or high (> 29.9) BMI. Klein reported a slight increase in risk of wet AMD associated with low and high BMI (RR 1.22 (95% CI 0.36–4.19); RR 1.27 (95% CI 0.65–2.48) respectively), however these were not significant over the 5-year follow up.

#### Smoking

Two studies [24, 27] reported smoking as a risk factor for wet AMD. Jonasson et al. [24] reported no increased risk in either past or current smokers, compared to those who have never smoked over a 5 year follow up period in 2,868 participants. In contrast, Tan et al. [27] also reported on current and past smokers compared to those who have never smoked in 2,454 participants over a 10-year period. There was a trend of increased risk of AMD in current smokers, however this was not significant (RR 1.9; 95% CI 0.6–5.3). They report no increased risk in those who were past smokers [27].

#### Blood pressure

Klein et al. [25] reported systolic blood pressure, diastolic blood pressure and pulse, and their association with wet AMD over a 5-year study period in 2,764 participants. There was a significantly increased risk of developing wet AMD for every 10mmHg increase of systolic blood pressure (RR 1.22; 95% CI 1.06–1.41), or every 10mmHg increase in pulse pressure (RR 1.34; 95% CI 1.14–1.60). Diastolic blood pressure was not associated with an increased risk.

#### Serum cholesterol

Three studies reported on serum cholesterol [24–26]. Results were reported as increase of 1mmol/L [24], per 10mmol/L [23], and per 1 SD increase [26]. Merle et al. [26] reported a slight increased risk, however this was not significant. The two other studies [24, 25] did not report any significant increased risk for AMD associated with serum cholesterol.

#### Serum HDL

Two studies reported the association between serum HDL and wet AMD [24, 25]. Jonasson et al. [24] reported HDL as an increase per mmol/L and Klein et al. [25] as an increase per 10mmol/L. Jonasson et al. [24] reported a decreased risk of wet AMD per mmol/L increase of HDL (HR 0.67; 95% CI 0.32–1.40), however this was not significant. Klein et al. [25] reported a slight decreased risk of wet AMD associated with HDL (HR 0.94; 95% CI 0.78–1.13), however this was also not significant.

#### Serum triglycerides

Merle et al. [26] were the only authors to report serum triglycerides and wet AMD. They report a non-significant increased risk of wet AMD per SD increase (HR 1.39; 95% CI 0.87, 2.23).

#### Diabetes mellitus

Jonasson et al. [24] were also the only study to look at diabetes mellitus as a potential independent risk factor for wet AMD. Having diabetes mellitus was associated with a slight and non-significant increased risk of wet AMD over the 5-years.

#### C reactive protein

Jonasson et al. [24] evaluated the potential of C-reactive protein as a risk factor over a 5-year study period. A C-reactive protein level of > 3mg/L was associated with a non-significant increase in risk of wet AMD when compared to the reference level of < 1mg/L. Levels of 1-3mg/L were not associated with an elevated risk of wet AMD.

#### Alcohol intake

Two studies [14, 22] reported alcohol intake and wet AMD; however, they reported alcohol intake differently. Cho et al. [22] reported alcohol intake as g/day. They did not find any significantly increased risk of wet AMD associated with alcohol intake. Knudtson et al. [14] reported alcohol intake as ‘never heavy drinking’, ‘past heavy drinking’ and ‘currently heavy drinking’, with heavy drinking defined as ‘4 or more drinks daily’, and a drink defined as’12-oz beer, 4-oz glass of wine or 1.5 oz of liquor’. They reported a non-significant increased risk of wet AMD following heavy drinking, both past and current over the 15-year study period in 3,509 participants.

#### Vitamins

Cho et al. [23] was the only study to report vitamins A, C and E as risk factors. There was no significant risk reported with any of the vitamins.

#### Beta-carotene

Two studies [23, 26] reported beta-carotene as a risk factor. Cho et al. [23] reported beta-carotene as distinct quartiles, while Merle et al. [25] reported it as increase per standard deviation. Cho et al. [23] reported a slight decreased risk in each quartile compared to the reference of quartile 1, which was the lowest beta-carotene measure, however none of these were significant. Merle et al. [26] reported a slight increased risk per 1 SD increase in beta-carotene levels, however this was also not significant.

#### Systematic review summary

There was a significantly increased risk of developing wet AMD with an increased systolic blood pressure and pulse pressure. While there were trends that current smoking, high or low BMI, heavy alcohol drinking, high C-reactive protein levels (> 3mg/L), diabetes mellitus and increase in serum triglycerides are associated with an increased risk of developing wet AMD, none of these associations were significant. Many of the studies were over a 5-year period and only a small number of participants developed wet AMD. These associations may become significant over a longer study period.

## Discussion – Results in context

We assessed risk factors and their association with the SHHEC cohort and conducted a systematic review of long-term (> 5 year) cohort studies assessing risk factors of wet AMD. Of the 18,107 subjects enrolled in SHHEC, 231 were treated for wet AMD in hospital over the 30-year follow up period. Unsurprisingly, higher age on enrolment was the risk factor most associated with increased risk of wet AMD within the SHHEC cohort. Smoking more than 5 cigarettes a day was significantly associated with increased risk of developing AMD, while vitamin K were significantly associated with reduced risk of developing AMD.

Within the systematic review, only seven studies were included in the final review; most studies reporting AMD risk factors were cross sectional, case control or retrospective studies which do not allow for a true view of directional risk (i.e. whether the risk or the disease was present first). Of the prospective cohort studies published, many used different methods for rating AMD, including early, intermediate, late (mixed wet and dry), geographic atrophy and any stage. While these provide helpful insights, they cannot be combined with our data that focuses solely on wet treated AMD.

Body weight: We found no evidence of an association between body weight and development of wet AMD within the SHHEC cohort. The two studies included in this review also found no significant association between body weight and wet AMD [24, 25]. A meta-analysis published in 2016 [12] found a significant association with obesity and late AMD, however it is not clear whether this was only wet AMD, or mixed (both wet and dry types). The authors theorized that this association may be due to fat-soluble xanthophyll carotenoids, such as lutein and zeaxanthin being absorbed into the adipose, rather than being available to the macula [12]. As our study was well powered and over a long duration, we believe body weight is not a factor for wet AMD.

Smoking: Within the SHHEC cohort, smoking itself (yes/no) and smoking more than 5 cigarettes a day were strongly associated with increased incidence of developing wet AMD. The two studies included in the systematic review assessing smoking followed participants for 5 [24] and 10 years [27]. The longer of the two studies did show a trend to increased risk in current smokers, however this was not significant. Our study follow up was longer and it is likely that the deleterious effect of smoking increases over time. Further, there were only 109 cases of wet AMD reported within these two studies, less than half of our study, and the larger SHHEC numbers may have made detection of the effect easier.

Smoking and its association with AMD has been investigated many times over the past 20 years, but the strength of the SHHEC study is not only the length of follow up and numbers of patients, but that surrogate markers for smoking (Cyanocobalamin, CO) captured all smokers. Several reviews have been published showing a significant effect of smoking on the development of AMD, however they are a mix of study designs [8, 28, 29]. Smoking causes oxidative stress, antioxidant depletion, complement activation, blood rheology and coagulation factors, and vascular changes which can result in AMD, and particularly the development of late AMD [30–32].

Blood pressure: We found no significant effect from blood pressure changes within the SHHEC cohort, however Klein et al. [25] reported an increased risk of developing wet AMD with increasing systolic blood pressure and pulse pressure. This could be explained by the fact that the BPs measured in SHHEC were on enrolment and were within the (relatively) normal range and were not repeated over the years. Thus, hypertension cannot be excluded as a shorter-term risk for AMD.

Serum cholesterol/triglycerides: We did not find any significant association between serum cholesterol or triglycerides within SHHEC nor did the three studies included in the review [24–26]. The previous published evidence including both types of AMD for cholesterol, triglycerides and associated lipoproteins is mixed, with some studies reporting an increased risk of AMD [33], and further meta-analyses finding no association [34].

Diabetes mellitus: Diabetes mellitus is associated with several eye conditions, such as diabetic retinopathy [35]. One study in the systematic review reported diabetes mellitus as a risk factor [24] for AMD. A meta-analysis assessing diabetes mellitus and varying stages of AMD in a mix of study designs found a significant association between late AMD and diabetes mellitus [36]. The authors [36] also report a significant association between diabetes mellitus and wet AMD in cross-sectional and case–control studies, but not cohort studies. However, many of the studies included in the meta-analysis were not adjusted for important risk factors such as smoking. Nevertheless diabetes mellitus does appear in most studies to be a risk for AMD. However interestingly within the SHHEC cohort, AMD was not significantly associated with previous diabetes mellitus diagnosis.

Inflammation and immunity: Within the SHHEC data, we found no association between inflammation and immunity risk factors and wet AMD. One study included in our review found a non-significant increased risk above 3mg/L. These results indicate that these risk factors are not associated with the development of AMD.

Alcohol intake: The studies included in the review reported no significant effect, except with “heavy” drinking. A meta-analysis assessing both early and late AMD and alcohol intake found a significant association between heavy drinking and early AMD, however they did not find any conclusive association with late AMD [11]. We found no association between heavy alcohol consumption and wet AMD within the SHHEC cohort.

Vitamins: Within the food data in SHHEC, the vitamin content of food was calculated from food frequency questionnaires. Vitamin K was found to have a protective effect against wet AMD within SHHEC. There has been little published about vitamin K and the association with AMD, with most research published around blood clotting [37]. Vitamin K has anti-inflammatory and anti-oxidative properties and therefore potentially an important vitamin for preventative treatment of AMD [38]. Vitamin K prevents excess vascular calcification, and this may explain its protective effect [39].

Antioxidants: Beta-carotene has long been thought to be associated with prevention of AMD, however, evidence suggests that there is little to no benefit from higher levels of beta-carotene in preventing AMD [40]. Within the SHHEC cohort and the review, we did not find any significant evidence that beta-carotene affects development of wet AMD, although SHHEC measures showed a slight non-significant decrease in risk, as did Cho 2004 [23], however Merle 2021 [24] found a slight increased risk. This suggests that beta-carotene levels are unlikely to be associated with AMD development or prevention at physiological levels.

### Strengths

SHHEC is a long-term study (> 30y), with secure data linkage for the 18,107 participants within the cohort. The risk factors that do link with AMD are thus present at least 30 years before AMD onset and are thus truly predictive, allowing monitoring, early management, aggressive smoking cessation policies, and perhaps earlier treatment in metabolic disease/prediabetes in the future. Further this was a large prospective study with many measured risk factors. Within the systematic review, we used strict inclusion and exclusion criteria to ensure a high degree of quality within the papers chosen.

### Limitations

Due to the strict inclusion criteria, we were not able to do a meta-analysis as there were not enough prospective cohort studies assessing risk factors and wet AMD. Many of the risk factors were measured in different ways, therefore not allowing direct comparison of risk ratios. While personal risk factors and some blood measures within SHHEC are extensive, some risk factors within SHHEC have missing data, which may impact our analyses. Furthermore, the only data on risk factors within SHHEC were taken at baseline, and thus we cannot say if any of our measures would have changed over time in the AMD cohort and habits change over time (i.e. smoking, alcohol intake).

Diet data was self-reported which may not be a reliable predictor of continued dietary habits, particularly over the 30-year period as diets and food “fads” change. Nevertheless, the long-term nature of the study with reasonable numbers developing the disease, allows robust assessment of risk factors present 30 years before AMD occurs clinically.

## Conclusions

While there is a large body of published work assessing risk factors and the development of AMD, only a small number assesses the development of wet AMD in a prospective cohort study. Meta-analyses have been published on the various risk factors of AMD over the past 20 years, however they use mixed study designs, and mixed AMD outcomes. We have added our own data to this body of work, providing evidence that increasing age and smoking is a high-risk factor for the development of wet AMD, while vitamin K is associated with reduced risk of wet AMD.

## Supplementary Information

Supplementary Material 1.

Supplementary Material 2.

## Data Availability

The data that support the findings of this study are owned and maintained by eDRIS. Data are available following a successful PBPP application to eDRIS, following their guidelines. To apply to eDRIS to access for data, contact phs.edris@phs.scot.

## Abbreviations

AMD: Age-related macular degeneration
SHHEC: Scottish Heart Health Extended Cohort
HR: Hazard Ratio
CI: Confidence Interval
BMI: Body mass index
anti-VEGF: Anti-vascular endothelial growth factor
SHHS: Scottish Heart Health Study
MONICA: Scottish MONICA study
CHI: Community Health Index
SMR01: Scottish Morbidity Record 01
ICD10: International Classification of diseases 10th edition
PRISMA: Preferred Reporting Items for Systematic Reviews and Meta-Analyses
PROSPERO: Prospective Register of Systematic Reviews
gamma GGT: Gamma glutamyl transferase
HDL: High density Lipoprotein
LDL: Low density lipoprotein
