# Supplementary material for: Risk factors for wet macular degeneration: a systematic review, with novel insights from the Scottish Heart Health Extended Cohort
Source: BMC Ophthalmol. 2025 Feb 10;25:67. doi: 10.1186/s12886-025-03868-5 (PMC11809110; doi:10.1186/s12886-025-03868-5)
Supplement: Supplementary file 1 — Supplementary Material 1. [file 12886_2025_3868_MOESM1_ESM.pdf]

Supplemental file 1. List of risk factors studied in SHHEC.

Table S1. Cohort characteristics on recruitment for SHHEC. N=18107

Variable	Quartile groups	Mean	Median	Missing
Age	Q1 ≤43.0<br>Q2 = 43.1-49.7<br>Q3= 49.8-55.8<br>Q4 ≥55.9	49.2	49.8	0
Education years	Group 1= < 9<br>Group 2= 10-19<br>Group 3= >20	11.11	10.00	397
Sex (ref: male)	M=8,862<br>F=9,245	-		0
Family CHD (ref: no)	N= 12,717<br>Y= 5,390	-		0
SIMD	Q1≤10.9<br>Q2=11.0-22.1<br>Q3=22.2-40.2<br>Q4 ≥40.3	27.8	22.2	0
Height (m)	Q1 ≤1.58<br>Q2=1.59-1.65<br>Q3=1.66-1.72<br>Q4 ≥1.73	1.66	1.66	13
Weight (kg)	Q1 ≤61<br>Q2=62-69<br>Q3=70-79<br>Q4 >80	71.71	70.00	14
BMI (kg/m2)	Q1 ≤22.9<br>Q2=23.0-25.3<br>Q3=25.4-27.9<br>Q4 >28.0	25.91	25.39	17
Waist circumference (cm)	Q1 ≤75.9<br>Q2=76.0-84.9<br>Q3=85.0-93.9<br>Q4 >94.0	85.82	85.00	12974
Current smoker	N= 9,759<br>Y= 8,348	-	-	0
Cigarettes/day	Group 1= 0<br>Group 2= 1-5<br>Group 3= 5-10<br>Group 4=10-19<br>Group 5 >20	7.784	0.00	0
Expired Carbon monoxide (ppm)	Q1 ≤1.9<br>Q2=2.0-2.9<br>Q3=3.0-14.9<br>Q4 ≥15.0	9.774	3.00	554
Fibrinogen (g/L)	Q1 ≤2.29<br>Q2=2.30-2.69	2.824	2.693	3089

	Q3=2.70-3.18<br>Q4 ≥3.19			
Cotinine (ng/ml)	Q1 ≤0.49<br>Q2=0.5-4.2<br>Q3=4.3-233.0<br>Q4 ≥233.1	116.41	4.32	5446
Thiocyanate (µlmol/L)	Q1 ≤32.9<br>Q2=33.0-50.6<br>Q3=50.7-110.0<br>Q4 ≥110.1	72.989	50.744	2331
Systolic blood pressure (mmHg)	Q1 ≤117<br>Q2=118-128<br>Q3=129-143<br>Q4 ≥144	132.1	129.0	10
Diastolic blood pressure (mmHg)	Q1 ≤72<br>Q2=73-80<br>Q3=81-88<br>Q4 ≥89	81.15	81.00	11
Pulse pressure (mmHg)	Q1 ≤40<br>Q2=41-48<br>Q3=49-58<br>Q4 ≥59	50.93	49.00	10
Pulse rate (mmHg)	Q1 ≤67<br>Q2=68-75<br>Q3=76-83<br>Q4 ≥84	77.33	76.00	93
Diabetes	N= 17,777<br>Y= 330		-	0
Glucose (mmol/L)	Q1 ≤4.31<br>Q2=4.32-4.75<br>Q3=4.76-5.27<br>Q4 ≥5.28	5.061	4.810	3370
Insulin (µU/mL)	Q1 ≤3.8<br>Q2=3.9-6.4<br>Q3=6.5-11.7<br>Q4 ≥11.8	10.03	6.50	4036
c-peptide (mg/mL)	Q1 ≤1.5<br>Q2=1.6-2.2<br>Q3=2.3-3.4<br>Q4 ≥3.5	2.74	2.29	3938
Total Cholesterol (mmol/L)	Q1 ≤5.3<br>Q2=5.4-6.1<br>Q3=6.2-7.0<br>Q4 ≥7.1	6.305	6.215	1819
HDL (mmol/L)	Q1 ≤1.15<br>Q2=1.16-1.41<br>Q3=1.42-1.71<br>Q4 ≥1.72	1.473	1.421	2692
Non-HDL (mmol/L)	Q1 ≤3.8<br>Q2=3.9-4.6<br>Q3=4.7-5.5<br>Q4 ≥5.6	4.827	4.722	2699

LDL (mmol/L)	Q1 ≤1.8<br>Q2=1.9-2.4<br>Q3=2.5-3.1<br>Q4 ≥3.2	2.683	2.457	5723
Triglycerides (mmol/L)	Q1 ≤1.0<br>Q2=1.1-1.5<br>Q3=1.6-2.3<br>Q4 ≥2.4	1.964	1.603	1836
Apolipoprotein-A (g/L)	Q1 ≤1.2<br>Q2=1.3-1.4<br>Q3=1.5-1.6<br>Q4 ≥1.7	1.558	1.530	3356
Apolipoprotein-B (g/L)	Q1 ≤0.8<br>Q2=0.9-1.0<br>Q3=1.1-1.2<br>Q4 ≥1.3	1.16	1.13	3356
Lipoprotein (a) (g/L)	Q1 ≤5.1<br>Q2=5.2-11.2<br>Q3=11.3-28.5<br>Q4 ≥28.6	20.5	11.3	3465
High sensitivity C reactive protein (mg/L)	Q1 ≤0.5<br>Q2=0.6-1.3<br>Q3=1.4-3.0<br>Q4= ≥3.1	2.915	1.410	3379
Homocysteine (µmol/L)	Q1= ≤11.3<br>Q2=11.4-13.5<br>Q3=13.6-16.5<br>Q4 ≥16.6	15.05	13.65	3383
Ferritin (µmol/L)	Q1= ≤34.1<br>Q2=34.2-73.6<br>Q3=73.7-136.1<br>Q4= ≥136.2	10.6.1	73.7	3989
HsTroponinI (pg/mL)	Q1 ≤1.8<br>Q2=1.9-3.9<br>Q3=4.0-6.0<br>Q4 ≥6.1	5.514	4.000	4948
NT-pro BNP (pg/mL)	Q1 ≤24.2<br>Q2=24.3-49.0<br>Q3=49.1-93.5<br>Q4 ≥93.6	92.43	49.11	6097
250HD raw (nmol/L)	Q1 ≤26.6<br>Q2=26.7-36.3<br>Q3=36.4-51.5<br>Q4 ≥51.6	41.491	36.417	4883
250HD adjusted (nmol/L)	Q1 ≤28.8<br>Q2=28.9-37.9<br>Q3=38.0-50.1<br>Q4 ≥50.2	38.046	41.491	4883
Vitamin B12 (pg/mL)	Q1 ≤306.9<br>Q2=307.0-393.9<br>Q3=394.0-504.6<br>Q4 ≥504.7	424.6	504.8	3953

gamma glutamyl transferase (units/L)	Q1 ≤14.9<br>Q2=15.0-21.6<br>Q3=21.7-34.8<br>Q4 ≥34.9	32.93	21.70	3532
Uric acid (mmol/L)	Q1 ≤229.2<br>Q2=229.3-277.0<br>Q3=227.1-331.1<br>Q4 ≥331.2	284.07	277.13	1811
Creatinine(mmol/L)	Q1 ≤79.1<br>Q2=79.2-87.6<br>Q3=87.7-96.7<br>Q4 ≥96.8	0.873	0800	3359
Cystatin-C (mg/L)	Q1 ≤0.63<br>Q2=0.64-0.69<br>Q3=0.70-0.79<br>Q4 ≥0.80	0.742	0.700	3354
Soluble FMS-like tyrosine kinase-1 (sFlt-1) (pg/ml)	Q1 ≤ 226.6<br>Q2 = 226.7-252.7<br>Q3= 252.8-283.5<br>Q4 ≥283.6	281.2	252.8	15800
TIMP metallopeptidase inhibitor 1 (TIMP1) (pg/ml)	Q1 ≤ 85.5<br>Q2 = 85.6-96.1<br>Q3 = 96.2-110.3<br>Q4 ≥ 110.4	100.44	96.23	15822
Placental Growth Factor (PGF) (pg/ml)	Q1 ≤ 13.3<br>Q2 = 13.4-15.8<br>Q3 = 15.9-19.0<br>Q4 = ≥19.1	18.09	15.90	15783
D-Dimer (pg/mL)	Q1 ≤ 104.9<br>Q2 = 105.0-183.9<br>Q3 = 184.0-280.9<br>Q4 ≥ 281.0	242.5	184.0	15768
Fat (g/d)	Q1 ≤ 63.7<br>Q2 = 63.8-79.9<br>Q3 = 80.0-97.9<br>Q4 ≥ 98.0	82.88	79.90	638
Saturated Fat (g/d)	Q1 ≤ 26.8<br>Q2 = 26.9-34.7<br>Q3 = 34.8-43.9<br>Q4 ≥ 44.0	36.38	34.70	882
Polyunsaturated fat (g/d)	Q1 ≤ 7.1<br>Q2 = 7.2-9.6<br>Q3 = 9.7-13.1<br>Q4 ≥ 13.2	10.73	9.60	882
Protein (g/d)	Q1 ≤ 67.4<br>Q2 = 67.5-79.5<br>Q3 = 79.6-93.6<br>Q4 ≥ 93.7	82.07	79.60	163
Carbohydrates(g/d)	Q1 ≤ 178.1<br>Q2 = 178.2-230.1<br>Q3 = 230.2-293.1<br>Q4 ≥ 293.2	242.6	230.2	218

Starch (g/d)	Q1 ≤ 105.9<br>Q2 = 105.9-140.0<br>Q3 = 140.0-181.7<br>Q4 ≥ 181.7	148.3	140.0	151
Sugar (g/d)	Q1 ≤ 61.7<br>Q2 = 61.8-84.7<br>Q3 = 84.8-117.5<br>Q4 ≥ 117.6	94.3	84.8	193
Alcohol (g/d)	Q1 = 0.0<br>Q2 = 0.1-6.6<br>Q3 = 6.7-18.9<br>Q4 ≥ 19.0	14.08	6.70	201
Cereal fibre (g/d)	Q1 ≤ 5.3<br>Q2 = 5.4-7.9<br>Q3 = 8.0-11.4<br>Q4 ≥ 11.5	9.103	8.000	121
Vegetable fibre (g/d)	Q1 ≤ 8.2<br>Q2 = 8.3-10.9<br>Q3 = 11.0-13.8<br>Q4 ≥13.9	11.29	11.00	197
Vitamin C (g/d)	Q1 ≤ 36.3<br>Q2 = 36.4-51.0<br>Q3 = 51.1-70.7<br>Q4 ≥ 70.8	56.02	51.10	199
Total energy (kcal/d)	Q1 ≤ 1629.5<br>Q2 = 1629.6-1996.1<br>Q3 = 1996.2-2443.3<br>Q4 ≥ 2443.4	2083	1996	373
Cholesterol (mg/d)	Q1 ≤ 253.9<br>Q2 = 254.0-331.1<br>Q3 = 331.2-425.0<br>Q4 ≥ 425.1	350.7	331.2	664
Retinol (mcg/d)	Q1 ≤ 380.3<br>Q2 = 380.4-585.5<br>Q3 = 585.6-880.7<br>Q4 ≥ 880.8	678.9	585.6	627
Beta carotene (mg/d)	Q1 ≤ 1648.0<br>Q2 = 1648.1-3140.7<br>Q3 = 3140.8-4573.4<br>Q4 ≥ 4573.5	3326.9	3140.8	1005
Alpha tocopherol (mg/d)	Q1<≤4.0<br>Q2= 4.1-5.4<br>Q3= 5.5-7.9<br>Q4 ≥ 8.0	7.086	5.500	510
Linoleic acid (mg/d)	Q1 ≤ 5.2<br>Q2 = 5.3-7.3<br>Q3 = 7.4-10.3<br>Q4 ≥ 10.4	8.518	7.400	575
Iron (mg/d)	Q1 ≤ 9.3<br>Q2 = 9.4-11.5<br>Q3 = 11.6-14.4<br>Q4 ≥ 14.5	12.43	11.60	413

Vitamin K (mg/d)	Q1 ≤ 43.8<br>Q2 = 43.9-60.7<br>Q3 = 60.8-81.6<br>Q4 ≥ 81.7	62.95	60.80	1920
Trans fatty acids (g/d)	Q1 ≤ 4.6<br>Q2 = 4.7-6.1<br>Q3 = 6.2-8.0<br>Q4 ≥ 8.1	6.687	6.200	4092
Vegetable trans Fatty acids (g/d)	Q1 ≤ 1.7<br>Q2 = 1.8-2.4<br>Q3 = 2.5-3.3<br>Q4 ≥ 3.4	2.698	2.500	4091
Animal trans Fatty acids (g/d)	Q1 ≤ 1.9<br>Q2 = 2.0-3.4<br>Q3 = 3.5-5.2<br>Q4 ≥ 5.3	3.991	3.500	4092

Supplementary Table 2. Analyses of Inflammation and immunity and wet AMD in SHHEC. Results are presented as Hazard Ratios (HR) with 95% Confidence Intervals (CI), and P values. Two models presented: no adjustment; age, sex, smoking, BMI and alcohol adjusted. Continuous data grouped as quartiles, or as specifically defined groups (i.e. education etc).

Variable	Grouping	Macular degeneration		Adjusted for + age + sex + smoking + BMI + alcohol	
		HR (95% CI)	P value	HR (95% CI)	P value
Ferritin (µmol/L)	Q1= ≤34.1	Reference		Reference	
	Q2=34.2-73.6	1.12 (0.75-1.67)	0.582	0.90 (0.60-1.35)	0.625
	Q3=73.7-136.1	1.03 (0.68-1.55)	0.899	0.77 (0.50-1.20)	0.249
	Q4= ≥136.2	1.18 (0.79-1.78)	0.415	0.93 (0.59-1.48)	0.770
Fibrinogen (g/L)	Q1 ≤2.29	Reference		Reference	
	Q2=2.30-2.69	0.80 (0.53-1.22)	0.301	0.67 (0.44-1.02)	0.065
	Q3=2.70-3.18	1.23 (0.85-1.79)	0.280	0.89 (0.61-1.30)	0.544
	Q4 ≥3.19	1.50 (1.02-2.20)	0.037	0.94 (0.63-1.40)	0.761
250HD raw (nmol/L)	Q1 ≤26.6	Reference		Reference	
	Q2=26.7-36.3	1.33 (0.86-2.05)	0.197	1.39 (0.90-2.15)	0.140
	Q3=36.4-51.5	1.05 (0.67-1.66)	0.822	1.13 (0.71-1.80)	0.600
	Q4 ≥51.6	1.33 (0.86-2.04)	0.201	1.55 (0.99-2.42)	0.054
250HD adjusted (nmol/L)	Q1 ≤28.8	Reference		Reference	
	Q2=28.9-37.9	1.09 (0.72-1.67)	0.675	1.15 (0.75-1.77)	0.514
	Q3=38.0-50.1	1.07 (0.70-1.64)	0.753	1.23 (0.80-1.89)	0.349
	Q4 ≥50.2	0.99 (0.64-1.52)	0.953	1.18 (0.76-1.84)	0.464
Vitamin B12 (pg/mL)	Q1 ≤306.9	Reference		Reference	
	Q2=307.0-393.9	0.90 (0.61-1.33)	0.598	0.94 (0.64-1.39)	0.773
	Q3=394.0-504.6	0.80 (0.54-1.19)	0.273	0.86 (0.58-1.29)	0.467
	Q4 ≥504.7	0.79 (0.52-1.19)	0.254	0.82 (0.54-1.25)	0.361
Hs C reactive protein (mg/L)	Q1 ≤0.5	Reference		Reference	
	Q2=0.6-1.3	1.07 (0.72-1.61)	0.732	0.89 (0.59-1.34)	0.579
	Q3=1.4-3.0	1.58 (1.08-2.32)	0.018	1.15 (0.77-1.73)	0.492
	Q4= ≥3.1	1.34 (0.88-2.04)	0.168	0.97 (0.62-1.53)	0.904
Hs C reactive protein (mg/L)	1mg/L increase	1.01 (0.98-1.04)	0.460	1.00 (0.97-1.03)	0.914

Supplementary table 3. Analyses of liver and kidney risk factors and wet AMD in SHHEC. Results are presented as Hazard Ratios (HR) with 95% Confidence Intervals (CI), and P values. Two models presented: no adjustment; age, sex, smoking, BMI and alcohol adjusted. Continuous data grouped as quartiles, or as specifically defined groups (i.e. education etc).

Variable	Grouping	Macular degeneration		Adjusted for + age + sex + smoking + BMI + alcohol	
		HR (95% CI)	P value	HR (95% CI)	P value
gamma glutamyl transferase (units/L)	Q1 ≤14.9	Reference		Reference	
	Q2=15.0-21.6	1.12 (0.77-1.64)	0.546	1.03 (0.70-1.53)	0.869
	Q3=21.7-34.8	1.33 (0.92-1.92)	0.135	1.30 (0.86-1.96)	0.203
	Q4 ≥34.9	1.00 (0.66-1.50)	0.986	1.06 (0.67-1.69)	0.796
Uric acid (mmol/L)	Q1 ≤229.2	Reference		Reference	
	Q2=229.3-277.0	1.04 (0.73-1.49)	0.811	0.89 (0.62-1.30)	0.552
	Q3=227.1-331.1	0.88 (0.60-1.29)	0.505	0.79 (0.52-1.22)	0.291
	Q4 ≥331.2	0.89 (0.60-1.32)	0.570	0.87 (0.54-1.41)	0.580
Creatinine (mmol/L)	Q1 ≤79.1	Reference		Reference	
	Q2=79.2-87.6	1.19 (0.82-1.72)	0.374	1.08 (0.74-1.59)	0.686
	Q3=87.7-96.7	0.80 (0.53-1.22)	0.305	0.77 (0.49-1.24)	0.284
	Q4 ≥96.8	0.95 (0.64-1.43)	0.817	0.87 (0.53-1.42)	0.572
Cystatin-C (mg/L)	Q1 ≤0.63	Reference		Reference	
	Q2=0.64-0.69	1.10 (0.47-2.61)	0.823	0.93 (0.29-2.20)	0.863
	Q3=0.70-0.79	1.28 (0.85-1.93)	0.236	1.01 (0.66-1.53)	0.981
	Q4 ≥0.80	1.98 (1.36-2.88)	<0.001	1.18 (0.78-1.77)	0.434

Supplementary table 4. Analyses of coagulation and wet AMD in SHHEC. Results are presented as Hazard Ratios (HR) with 95% Confidence Intervals (CI), and P values. Two models presented: no adjustment; age, sex, smoking, BMI and alcohol adjusted. Continuous data grouped as quartiles, or as specifically defined groups (i.e. education etc).

Variable	Grouping	Macular degeneration		Adjusted for + age + sex + smoking + BMI + alcohol	
		HR (95% CI)	P value	HR (95% CI)	P value
Soluble FMS-like tyrosine kinase-1 (sFlt-1) (pg/ml)	Q1 ≤ 226.6	Reference		Reference	
	Q2 = 226.7-252.7	1.10 (0.27-4.39)	0.896	0.93 (0.23-3.76)	0.919
	Q3= 252.8-283.5	1.00 (0.22-4.49)	0.997	0.81 (0.18-3.75)	0.790
	Q4 ≥283.6	1.61 (0.40-6.43)	0.504	1.18 (0.27-5.13)	0.821
TIMP metallopeptidase inhibitor 1 (TIMP1) (pg/ml)	Q1 ≤ 85.5	Reference		Reference	
	Q2 = 85.6-96.1	1.06 (0.31-3.67)	0.925	0.70 (0.20-2.50)	0.588
	Q3 = 96.2-110.3	0.53 (0.10-2.71)	0.442	0.24 (0.04-1.34)	0.105
	Q4 ≥ 110.4	1.07 (0.26-4.50)	0.924	0.48 (0.10-2.20)	0.343
Placental Growth Factor (PGF) (pg/ml)	Q1 ≤ 13.3	Reference		Reference	
	Q2 = 13.4-15.8	0.78 (0.22-2.77)	0.702	0.62 (0.17-2.23)	0.463
	Q3 = 15.9-19.0	0.67 (0.17-2.70)	0.578	0.31 (0.07-1.32)	0.113
	Q4 = ≥19.1	0.61 (0.12-3.04)	0.546	0.26 (0.05-1.39)	0.115
D-Dimer (pg/ml)	Q1 ≤ 104.9	Reference		Reference	
	Q2 = 105.0-183.9	1.55 (0.26-9.29)	0.632	1.02 (0.17-6.21)	0.983
	Q3 = 184.0-280.9	3.06 (0.61-15.27)	0.172	1.86 (0.36-9.54)	0.456
	Q4 ≥ 281.0	2.28 (0.41-12.48)	0.344	1.14 (0.20-6.46)	0.886
