# Supplementary material for: Risk factors for wet macular degeneration: a systematic review, with novel insights from the Scottish Heart Health Extended Cohort
Source: BMC Ophthalmol. 2025 Feb 10;25:67. doi: 10.1186/s12886-025-03868-5 (PMC11809110; doi:10.1186/s12886-025-03868-5)
Supplement: Supplementary file 2 — Supplementary Material 2. [file 12886_2025_3868_MOESM2_ESM.docx]

Supplemental Table 5. Details for studies included in the systematic review. Details include: study ID, country, study name, size, follow up period, risk factors studied, outcomes, model adjustments and Newcastle-Ottowa Scale score.

| Author, Year, Country | Study size | Follow up period | Risk factor | Adjustments | Results | NOS score |
| --- | --- | --- | --- | --- | --- | --- |
| Cho 2000  USA | 32764 women  29488 men | 8-10 yrs;  Recruit 1976 (F) 1986 (M);  1992 FU | Alcohol g/day | Age, smoking | Cho et al assessed alcohol as g/day (0.1-4.9; 5-14.9; 15-29.9; 30+), compared to 0g/day. There was no association between alcohol (g/day) and wet AMD. | 4*  1*  3*  good |
| Cho 2004  USA | 77562 women,  121700 recruited;  40866 men, 51529 recruited; | 18 yr (women),  Recruit 1976;  1996 FU  12 yr (men),  Recruit 1986;  1996 FU | Vitamin A, Vitamin C, Vitamin E, beta-carotene, | Age, smoking, BMI, hypertension, physical activity, profession, high cholesterol, alcohol, energy & fish intake | Cho et al assessed vitamins A, C, E and beta carotene as quartiles, using Q1 as reference. There was no significant association between any risk factor assessed and wet AMD. | 4*  2*  3*  good |
| Jonasson 2014  Iceland | 2868;  4910 recruited; | 5 yr;  2002-6 recruit; 2007-11 FU | Smoking, diabetes, BMI, total cholesterol, HDL, C reactive protein | Age, sex, smoking, hypertension, cod liver oil, pulse pressure, diabetes, BMI, total cholesterol, HDL, C reactive protein | There was no increased risk of wet AMD associated with current (OR 0.97; 95% CI 0.39-2.42) or past smoking (OR 0.93; 95% CI 0.52-1.65). There was also no increased risk of wet AMD found per 1kg/m3 increase in weight (OR 0.96; 95% CI 0.89-1.04).  CRP was also not associated with wet AMD (1-3mg/L = OR 1.06; 95% CI 0.52-2.18); >3mg/L OR 1.31; 95% CI 0.60-2.86), ref <1mg/L). Diabetes was not associated with wet AMD (OR 1.29; 95% CI 0.56-2.98)  Serum cholesterol (OR 1.03; 95% CI 0.80-1.32) and HDL (OR 0.67; 95% CI 0.32-1.40) were not associated with wet AMD. | 4*  2*  2*  good |
| Klein 2003  USA | 2764;  4926 recruited;  3684 at 5YR FU;  2764 at 10yr FU | 10 yr;  1988-90 recruit;  1993-5 5YR FU;  1998-2000 10 YR FU | SBP, DBP, pul press, total cholesterol, HDL, BMI, | Age, sex, smoking, alcohol, vitamin use | An increase of 10mmHg in SBP (1.22; 95% CI 1.06–1.41) and pulse pressure (1.34; 95% CI 1.14–1.60) were associated with increased risk of wet AMD. DBP was not associated with an increased risk (0.95; 95% CI 0.70–1.29).  There was no increased risk for wet AMD associated with low BMI (<22) or high BMI (>30), compared to normal BMI (22-29.0) (1.22 (0.36–4.19); 1.27 (0.65–2.48) respectively).  Serum cholesterol (0.93; 95% CI 0.86–1.00) and HDL (0.94; 95% CI 0.78–1.13) were also not associated with AMD. | 4*  2*  3*  good |
| Knudtson 2007  USA | 3509 | 15 yr | Alcohol (heavy drinking – never, past, current) | Age, SBP, vitamin use, smoking, sex | Heavy drinking was not associated with wet AMD, in current heavy drinkers (3.00; 95% CI 0.67, 13.5) and past heavy drinkers (1.63; 95% CI 0.72, 3.71), however this was not significant. | 4*  1*  2*  good |
| Merle 2021  France | 963 recruited;  435 FU | Recruit 2006-8;  2015-17 FU | Beta-carotene, cholesterol, triglycerides | Sex, AMD grade, smoking, alcohol, season, BMI, diabetes, cholesterol, triglycerides, martial status, activity, genetic risk score | Serum cholesterol (HR 1.33; 95% CI (0.71, 2.47), triglycerides (HR 1.39; 95% CI 0.87, 2.23) and beta-carotene (HR 1.26; 95% CI 0.80, 1.99). (per 1SD increase) were not significantly associated with AMD per 1 SD increase. | 3*  2*  2*  good |
| Tan 2007  Australia | 2454  Recruit 3654 | 10 yr  Recruit: 1992-93  FU:2002-05 | Smoking (never, past current) | Age, sex, WBC, family history of AMD, HDL, very fair skin colour, fish intake | Current (1.9 (0.6-5.3) and past (0.8 (0.4-1.9) smoking was not associated with wet AMD (1.9 (95% CI 0.6-5.3); 0.8 (95% CI 0.4-1.9) respectively). | 4*  2*  3*  good |
